# Effect of heterospecific pollen deposition on pollen tube growth depends on the phylogenetic relatedness between donor and recipient

**DOI:** 10.1093/aobpla/plaa016

**Published:** 2020-06-05

**Authors:** Nathália Susin Streher, Pedro Joaquim Bergamo, Tia-Lynn Ashman, Marina Wolowski, Marlies Sazima

**Affiliations:** 1 Graduate Program in Plant Biology, University of Campinas, Campinas, SP, Brazil; 2 Department of Biological Sciences, University of Pittsburgh, Pittsburgh, PA, USA; 3 Graduate Program in Ecology, University of Campinas, Campinas, SP, Brazil; 4 Institute of Natural Sciences, Federal University of Alfenas, Alfenas, MG, Brazil; 5 Plant Biology Department, Institute of Biology, University of Campinas, Campinas, SP, Brazil

**Keywords:** Competition, facilitation, interspecific pollen transfer, pollen germination, pollinator sharing

## Abstract

Co-flowering plant species may interact via pollinators leading to heterospecific pollen transfer with consequences for plant reproduction. What determines the severity of heterospecific pollen effect on conspecific pollen performance is unclear, but it may depend on the phylogenetic relatedness of the interactors (pollen donors and recipient). The heterospecific pollen effect might also depend on the extent to which plants are exposed to heterospecific pollen over ecological or evolutionary timescales. For instance, generalist-pollinated plant species might tolerate heterospecific pollen more than specialists. Here, we tested whether heterospecific pollen effects are stronger between closely related species than phylogenetically distant ones in a tropical highland community. Then, based on these results, we determined whether responses to heterospecific pollen were stronger in generalized vs. specialized plant species. We applied heterospecific pollen from close (congeneric) or distant (different families) donors alone or with conspecific pollen on stigmas of three recipient species (one generalist, *Sisyrinchium wettsteinii*; and two specialists, *Fuchsia campos-portoi* and *Fuchsia regia*) and scored pollen tube performance in styles. In all species, pollen from closely related donors grew pollen tubes to the base of the style indicating a high potential to interfere with seed set. Conversely, distantly related heterospecific pollen had no effect on either specialist *Fuchsia* species, whereas enhanced performance of conspecific pollen was observed in generalist *S*. *wettsteinii*. The strong effect of phylogenetic relatedness of donor and recipient might have obscured the role of pollination specialization, at least for the three species examined here. Therefore, phylogenetic relatedness mediated the effect of heterospecific pollen on post-pollination success, with possible consequences for reproductive trait evolution and community assembly for further studies to explore.

## Teaser

Co-flowering species interact via pollinators leading to heterospecific pollen transfer (pollen from other species) with consequences for plant reproduction. We experimentally demonstrated that phylogenetic relatedness mediated the effect of heterospecific pollen on post-pollination success. Using three species as recipients, we showed that pollen donors of closely related species (same genus) affected pollen-tube growth and had a negative impact on the post-pollination success of the recipient species. However, when pollen donors were distantly related (different family), recipient species tolerated or were even benefited by growing more pollen tubes than without the presence of heterospecific pollen.

## Introduction

Most flowering plants rely on animal pollinators to transfer their pollen grains (Ollerton *et al.* 2011) to conspecific stigmas and set seeds. Plants sharing pollinators may compete or facilitate pollinator visits (i.e. pre-pollination interactions; Rathcke 1983; Moeller 2004; Mitchell *et al*. 2009). If two species exploit the same pollinator, interspecific pollinator movements can result in conspecific pollen (hereafter *CP*) loss and heterospecific pollen (hereafter *HP*) deposition on stigmas (i.e. post-pollination interactions) with a potential impact on the reproductive success of both (Morales and Traveset 2008; Ashman and Arceo-Gómez 2013; Moreira-Hernández and Muchhala 2019). Hence, plant–plant interactions via pollinators are traditionally interpreted as negative, at least for the donor’s perspective (male component), because loosing *CP* is always a waste of gametes that could otherwise affect conspecific reproduction (Waser 1978; Bell *et al.* 2005). Historically, *HP* receipt (female component) was also mainly interpreted as negative because loss of seed fitness is even more costly than loss of pollen grains (Rathcke 1983). However, once pollen grains are on heterospecific stigmas they can encounter diverse barriers that can lead pollen tubes to different fates (Swanson *et al.* 2004). So even though there is considerable evidence that *HP* can reduce recipient fitness (e.g. Da Silva and Sargent 2011; Briggs *et al.* 2015; Fonseca *et al.* 2016; Arceo-Gómez *et al.* 2018), there is also evidence that *HP* has no effect on recipient reproduction (e.g. Kohn and Waser 1985; Caruso and Alfaro 2000; Montgomery 2009). The lack of a consistent pattern may be related to the fact that the outcome of the interaction does not depend solely on the recipient, but also on interactive effects of recipient with *HP* donors (Arceo-Gómez *et al.* 2019). Assessing what underlies the prevalence and strength of *HP* effects on post-pollination success is essential to understand its role in shaping flowering communities.

Heterospecific pollen can impact recipient plants by physically blocking the stigma (Waser and Fugate 1986; Galen and Gregory 1989) and/or interfering with *CP* performance (Arceo-Gómez and Ashman 2011). The interference can be through allelopathic effects that retard *CP* tube or ovule growth (Sukhada and Chandra 1980; Thomson *et al.* 1981) or through *HP* fertilizing conspecific ovules (Harder *et al.* 1993; Burgess *et al.* 2008). The latter is most common among closely related species and can, ultimately, result in hybridization (Wendt *et al.* 2001; Wendt *et al.* 2008; Arceo-Gómez and Ashman 2011). As pollen–pistil interactions may be compatible between species with recent evolutionary history (Moreira-Hernández and Muchhala 2019), those with distant history face considerable morphological and genetical incongruities that preclude much *HP* development after deposited on stigmas (Hogenboom 1975). Thus, we predict *HP* effects will increase with decreasing recipient–donor relatedness (Ashman and Arceo-Gómez 2013; Arceo-Gómez and Ashman 2016). Moreover, intrinsic traits of recipients can mediate *HP* effects, such as its mating system since self-incompatible species possess stronger barriers than self-compatible ones to improper pollen growth (Harder *et al.* 1993; Ashman and Arceo-Gómez 2013). The degree to which recipients restrict pollinator accessibility to flowers could also correlate with tolerance to *HP* receipt. In this sense, generalist-pollinated species (i.e. that exploit a wide variety of pollinators; Ollerton *et al.* 2007) might not be impaired as much by *HP* as specialist-pollinated ones, because the former were presumably exposed more often and to higher and more diverse loads of *HP* over generations (Fang and Huang 2013; Arceo-Gómez *et al.* 2016; Fang *et al.* 2019). Donor traits like *HP* size and apertures (Ashman and Arceo-Gómez 2013), as well as extrinsic factors such as *HP* arrival time on stigmas (Suárez-Mariño *et al.* 2019), abiotic conditions (Celaya *et al.* 2015) and *HP* load diversity and identity (Arceo-Gómez and Ashman 2011) also are known to contribute on recipient’s post-pollination outcomes.

Species floral traits influence how plants exploit pollinators and consequently also affect the likelihood of *HP* transfer (Minnaar *et al.* 2019). For instance, species with specialized floral morphology constrain accessibility to only few pollinators that provide the best pollen transfer (Stebbins 1970; Ollerton *et al.* 2007), reducing the chance of *HP* deposition. Nevertheless, generalist-pollinated flowers are the majority in flowering communities (Waser *et al.* 1996; Memmott 1999; Olesen and Jordano 2002), making *HP* deposition a frequent phenomenon, even in spite of all mechanisms to control *HP* transfer (McLernon *et al.* 1996; Montgomery and Rathcke 2012; Fang and Huang 2013). But the effects vary between species and thus selection to avoid *HP* is expected to vary. Hence, species might possess mechanisms that act filtering only *HP* that is indeed detrimental to recipients (Ashman and Arceo-Gómez 2013) or even compensate for recipient losses by maximizing pollen dispersal to conspecifics (Muchhala *et al.* 2010). Moreover, the benefits of sharing pollinators with heterospecifics via facilitation may outweighs *HP* costs (Tur *et al*. 2016) indicating that species with overlapping pre-pollination mechanisms may be unaffected by receipt of each other’s pollen (Gross *et al.* 2000). Thus, to fully understand the magnitude of *HP* as an evolutionary force driving floral trait divergence or community assembly, we need to consider its costs on both female and male reproductive components as well as in a community context (Muchhala *et al.* 2010).

Here, we used an experimental approach to assess the post-pollination *HP* effects between sets of species with similar flowers that share pollinators in a tropical highland community. The species studied here were categorized as specialist- or generalist-pollinated depending on whether they were visited by one or more than one group of pollinators, respectively (see Bergamo *et al.* 2020a). The taxonomic classification of these species (genus and family) was used to establish pairs of closely related taxa (species in the same genus) vs. more distantly related (species in different families). Our main goal was to test whether the effects of *HP* differ with phylogenetic distance between donor and recipient. Because there are fewer post-pollination barriers between closely related species, we hypothesize that *HP* effects are stronger between closely related species than between phylogenetically distant ones (Ashman and Arceo-Gómez 2013; Arceo-Gómez and Ashman 2016). As we choose species with distinct pollination systems to address this question, we used the results from experiments to establish whether recipients respond differently depending on their likelihood of receiving *HP*. Since species pollinated by numerous animals can receive more *HP* (Fang and Huang 2013; Arceo-Gómez *et al*. 2016), we hypothesize that generalist-pollinated species are more able to tolerate the presence of *HP* than specialist-pollinated species, and thus suffer less impact by *HP* receipt.

## Materials and Methods

### Study site

The study was conducted in the plateau of the Itatiaia National Park (22°21′’S, 44°40′W) that is in the Atlantic forest domain, southeastern Brazil. Data collection was carried out between 2000 and 2400 m a.s.l. where the lower altitude spans montane forests and the higher is dominated by grasses, herbs and shrubs (known as *campos de altitude*; Vasconcelos 2011).

### Selection of studied species

**Figure 1.**
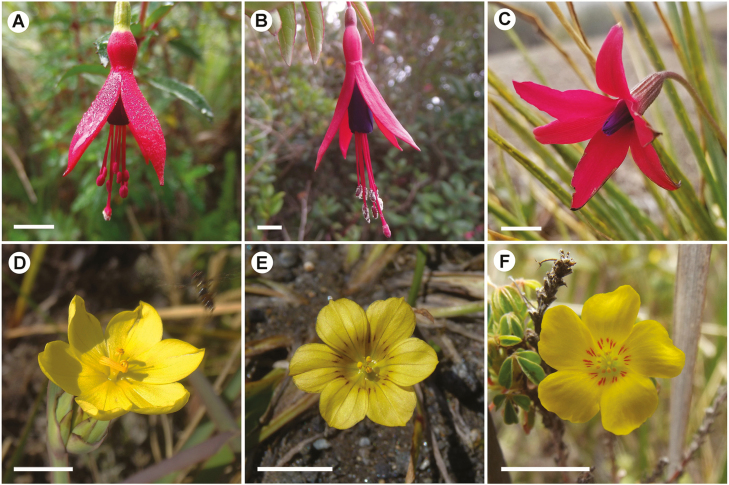
Flowers of species used in experiments of heterospecific pollen effects. (A) *Fuchsia campos-portoi*. (B) *Fuchsia regia*. (C) *Barbacenia gounelleana*. (D) *Sisyrinchium wettsteinii*. (E) *Sisyrinchium glaziovii*. (F) *Oxalis confertissima*. Bar = 0.5 cm.

We conducted hand pollination experiments with three trios of sympatric co-flowering species (Fig. 1; Table 1). Each trio was composed of (i) one pollen recipient species, (ii) one *HP* donor phylogenetically close to the recipient species and (iii) one *HP* donor phylogenetically distant but with phenotypically similar flowers. We considered congeners as phylogenetically close and species from different families as phylogenetically distant. Based on this assumptions, the recipients chosen included two *Fuchsia* hummingbird-pollinated species, considered as specialists (previously classified in Bergamo *et al.* 2020a): *Fuchsia campos-portoi*, that has 113.7 ± 20.83 (mean ± standard deviation) ovules per ovary and a stigmatic surface area of 0.01 cm^2^ and *F. regia*, that has 122.38 ± 25.23 (mean ± standard deviation) ovules per ovary and a stigmatic surface area of 0.05 cm^2^. The other recipient was *Sisyrinchium wettsteinii* that has 60.5 ± 16.8 (standard deviation) ovules per ovary, a stigmatic surface area of 0.001 cm^2^ and is visited by various insects (i.e. bees, flies and beetles), considered a generalist-pollinated species (previously classified in Bergamo *et al.* 2020a). Besides fitting the assumptions described before, these species were chosen to be recipients also due to their high flower abundance in field, their pollination system and the fact that they experience *HP* deposition naturally (e.g. 52 % of *F. campos-portoi*, 50 % of *F. regia* and 53 % of *S. wettsteinii* stigmas received *HP* in the field; N. S. Streher *et al*., unpubl. data). Species used as pollen donors were *Barbacenia gounelleana*, *Oxalis confertissima*, *Sisyrinchium glaziovii* and *F. campos-portoi* and *F. regia*. *Barbacenia gounelleana*, *S. glaziovii* and *O. confertissima* were used as pollen donors but not as recipients because they did not fit our requirements to be a recipient model in this study.

**Table 1.** Species used as pollen recipient in the experiments, their floral phenotypes and the identity of heterospecific pollen donors.

		*HP* donor species	
Pollen recipient species	Floral phenotype	Phylogenetically close	Phylogenetically distant
*Fuchsia campos-portoi* (Onagraceae)	Specialist (hummingbird-pollinated)	*Fuchsia regia* (Onagraceae)	*Barbacenia gounelleana* (Velloziaceae)
*Fuchsia regia* (Onagraceae)	Specialist (hummingbird-pollinated)	*Fuchsia campos-portoi* (Onagraceae)	*Barbacenia gounelleana* (Velloziaceae)
*Sisyrinchium wettsteinii* (Iridaceae)	Generalist (various insects)	*Sisyrinchium glaziovii* (Iridaceae)	*Oxalis confertissima* (Oxalidaceae)

### Experimental treatments

Five types of hand pollinations were conducted in the field: (i) outcross conspecific pollen (*CP*); (ii) heterospecific pollen from the phylogenetically distant species (*HP distant*); (iii) heterospecific pollen from the phylogenetically close species (*HP close*); (iv) mixture of outcross (*CP + HP distant*); (v) mixture of outcross (*CP + HP close*). Pollination by pure loads of *HP* was conducted to assess post-pollination barriers and to aid in the interpretation of the results of mixtures as pollen grains from congeners are usually hard to distinguish and once pollen tubes grow into the style species identity is unknown. Pistils were fixed in 50 % FAA (formalin-acetic acid-alcohol) solution (Johansen 1940) 24 h after hand pollinations since pilot experiment demonstrated that this was enough time for pollen tubes to reach style base in all species. Fixed materials were cleared with NaOH 9 N, heated at 60 °C for 20 min, stained with blue aniline and observed in a fluorescent microscope (Martin 1959). For each pistil, we counted the number of pollen grains deposited on stigmas, the number of pollen grains germinated in stigmas, pollen tubes at the tip and base of styles. Based on what we scored, we refer to *post-pollination success* as how many of the *CP* grains adhered to stigmas successfully develop further along the style.

Buds were bagged to avoid pollen contamination from visitors. Because anthers and stigmas in *S. wettsteinii* flowers are positioned close to each other we emasculated them in bud prior to experiments to avoid self-pollen contamination. Emasculation was not necessary for *Fuchsia* species because their flowers are protogynous (stigmas are receptive before anthers dehiscence preventing self-pollination). Pollen applied was fresh which means that recipient and donor species were flowering at the same time during experiments in the field. To standardize our method of pollen transfer we touched one anther per individual donor in each recipient stigma. We used three individuals as *CP* donors and only one as *HP* donor so mixed treatments had ca. 75 % *CP* and 25 % *HP* mix of pollen grains. Heterospecific pollen receipt in natural communities varies extensively, with most species receiving on average 20 % of *HP* (see Ashman and Arceo-Gomez 2013; Fang and Huang 2013). Because of that, we checked the *HP* naturally deposited in flowers of the species studied here during one flowering season. The *HP* deposition varied from 0–16 % in *F. campos-portoi*, 0–2 % in *F. regia* and 0–100 % in *S. wettsteinii* and the mean percentage of *HP* loads in flowers was 4 %, 0.8 % and 27 % for each species, respectively (N. S. Streher *et al.*, unpubl. data). Hence, the usual ratio 50:50 of *CP:HP* used in experiments seemed too high in general for these species (even for the generalist-pollinated *S. wettsteinii*), leading us to use the ratio 75:25. In these treatments, *CP* was applied first and *HP* immediately after. Differences in pollen size and pollen adherence capability among species may have influenced the final pollen load (see Table 2 for pollen load). To avoid self-incompatibility reactions, the individuals that were used as *CP* donors were always separated by at least 200 m from the recipient.

**Table 2.** Number of pollen grains applied in each treatment for each recipient species (mean ± standard deviation). The number of recipients used in each treatment are in parenthesis.

	Recipient		
Treatment	*Fuchsia campos-portoi*	*Fuchsia regia*	*Sisyrinchium wettsteinii*
*CP*	128.05 ± 45.55 (20)	293.81 ± 105.62 (22)	81.09 ± 51.21 (21)
*HP close*	135.913 ± 109.76 (23)	280.61 ± 97.20 (13)	52.34 ± 28.84 (41)
*CP + HP close*	164.58 ± 77.48 (24)	340.5 ± 147.48 (14)	80.67 ± 37.37 (28)
*HP distant*	83.15 ± 62.48 (19)	119.166 ± 176.58 (8)	8.68 ± 11.64 (32)
*CP + HP distant*	106.7 ± 45.63 (20)	200.76 ± 124.20 (14)	45.64 ± 34.59 (17)

### Statistical analysis

To evaluate the effect of pollination treatments on recipient post-pollination success, we fitted generalized linear mixed models (GLMMs) using binomial distributions in the glmmTMB package (Brooks *et al.* 2017) in R v. 3.5.1 (R Core Team 2018). We fitted models for each of the three response variables resulting in three models for each recipient species. The response variable of each model was a matrix containing the total pollen deposited in stigma and the pollen response resulting from this deposition in the different portions of the same pistil (pollen grains germinated in the stigma, pollen tubes at the tip of the style and pollen tubes at the base of the style). This model accounted for variation in the amount of pollen deposited on the stigmas. For all models, pollination treatments were the fixed effect and individuals were included as a random effect. Model assumptions were checked graphically. To verify the significance of each model, we compared the built models with a null model that included the respective response variable and only the intercept. Since we were specifically interested in comparing treatment effects relative to outcross *CP*, we performed a *post hoc* test using the package emmeans (Lenth *et al*. 2020).

## Results

Pollination treatments influenced recipient post-pollination success revealing different outcomes depending whether *HP* was present and its source. Pollen performance was explained by differences among treatments since all models performed better than the null models (Table 3). Model comparisons within each pistil portion, described below, were interpreted relative to *CP* treatment as this corresponds to the ideal situation for reproduction (i.e. only conspecific and no foreign pollen grains on stigmas; see Table 4 for more).

**Table 3.** Comparisons between built and null models explaining species responses to heterospecific pollen presence in the three portions of the pistil. AIC = Akaike Information Criteria used to estimate model performance; χ ^2^ = generalized linear mixed effect value for model’s comparison. Bold values indicate significant effects at *P* < 0.05.

Model	AIC	χ ^2^	*P*
A) *Fuchsia campos-portoi*			
Pollen grains germinated in the stigma			
* Treatment*	2665.4		
* Null*	5568.0	2912.7	**<0.001**
Pollen tubes at the tip of the style			
* Treatment*	1395.7		
* Null*	2592.7	1207	**<0.001**
Pollen tubes at the base of the style			
* Treatment*	685.6		
* Null*	1059.8	384.15	**<0.001**
B) *Fuchsia regia*			
Pollen grains germinated in the stigma			
*Treatment*	1976.4		
*Null*	4191.2	2224.8	**<0.001**
Pollen tubes at the tip of the style			
*Treatment*	1001.1		
*Null*	1677.3	686.29	**<0.001**
Pollen tubes at the base of the style			
*Treatment*	452.27		
*Null*	905.91	463.64	**<0.001**
C) *Sisyrinchium wettsteinii*			
Pollen grains germinated in the stigma			
* Treatment*	906.31		
* Null*	1817.06	920.75	**<0.001**
Pollen tubes at the tip of the style			
* Treatment*	893.61		
* Null*	2396.31	1512.7	**<0.001**
Pollen tubes at the base of the style			
* Treatment*	920.34		
* Null*	1754.90	844.56	**<0.001**

**Table 4.** Contrasts between outcross conspecific pollen (CP) and the other treatments. EMM = estimated marginal means; SE = standard error. Bold values indicate significant effects at *P* < 0.05.

Treatment	EMM	SE	*t*	*P*
A) *Fuchsia campos-portoi*				
Pollen grains germinated in the stigma				
*HP close*	**0.724**	**0.073**	**9.954**	**<0.0001**
*CP + HP close*	**0.881**	**0.067**	**13.199**	**<0.0001**
*HP distant*	**−0.806**	**0.108**	**−7.460**	**<0.0001**
*CP + HP distant*	**−1.028**	**0.102**	**−10.064**	**<0.0001**
Pollen tubes at the tip of the style				
*HP close*	**0.647**	**0.074**	**8.688**	**<0.0001**
*CP + HP close*	**0.768**	**0.066**	**11.596**	**<0.0001**
*HP distant*	**2.603**	**0.294**	**8.864**	**<0.0001**
*CP + HP distant*	−0.126	0.091	−1.375	0.645
Pollen tubes at the base of the style				
*HP close*	**0.441**	**0.120**	**3.678**	**0.003**
*CP + HP close*	**0.886**	**0.118**	**7.540**	**<0.0001**
*HP distant*	**4.103**	**0.551**	**7.443**	**<0.0001**
*CP + HP distant*	−0.284	0.116	−2.453	0.110
B) *Fuchsia regia*				
Pollen grains germinated in the stigma				
*HP close*	**−0.557**	**0.104**	**−5.371**	**0.013**
*CP + HP close*	**−1.546**	**0.099**	**−15.625**	**<0.0001**
*HP distant*	−0.319	0.161	−1.988	0.284
*CP + HP distant*	**−1.774**	**0.12**	**−14.837**	**<0.0001**
Pollen tubes at the tip of the style				
*HP close*	0.266	0.124	2.144	0.215
*CP + HP close*	0.24	0.114	2.098	0.233
*HP distant*	**2.453**	**0.460**	**5.333**	**<0.0001**
*CP + HP distant*	0.175	0.136	1.288	0.699
Pollen tubes at the base of the style				
*HP close*	0.633	1.167	0.543	0.982
*CP + HP close*	2.579	1.021	2.526	0.098
*HP distant*	2.769	1.461	1.895	0.331
*CP + HP distant*	−0.911	1.023	−0.891	0.899
C) *Sisyrinchium wettsteinii*				
Pollen grains germinated in the stigma				
*HP close*	**1.320**	**0.251**	**5.267**	**<0.0001**
*CP + HP close*	**2.358**	**0.251**	**9.379**	**<0.0001**
*HP distant*	**3.369**	**0.305**	**11.047**	**<0.0001**
*CP + HP distant*	−0.222	0.300	−0.738	0.947
Pollen tubes at the tip of the style				
*HP close*	**0.633**	**0.185**	**3.430**	**0.007**
*CP + HP close*	**1.603**	**0.199**	**8.059**	**<0.0001**
*HP distant*	**4.585**	**0.328**	**13.978**	**<0.0001**
*CP + HP distant*	**−1.506**	**0.259**	**−5.822**	**<0.0001**
Pollen tubes at the base of the style				
*HP close*	0.168	0.192	0.879	0.904
*CP + HP close*	0.004	0.183	0.022	1.000
*HP distant*	**3.759**	**0.588**	**6.396**	**<0.0001**
*CP + HP distant*	**−0.982**	**0.187**	**−5.252**	**<0.0001**

### Recipient *F. campos-portoi* (specialist-pollination)

**Figure 2.**
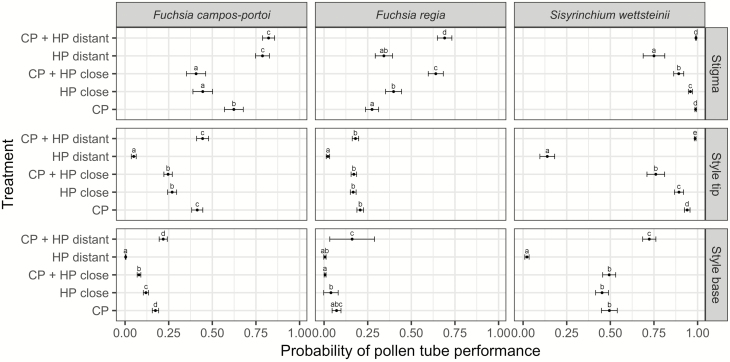
Probability of pollen performance of each treatment in the three portions of each recipient’s pistil (stigma, style tip and style base). In each block, different letters indicate significant differences at *P* < 0.05.

At the stigma, treatments containing *HP* from phylogenetically distant species showed greater probabilities of pollen germination (>78 %) compared to treatment containing only *CP* (*HP distant*, *t* = −7.460, df = 99, *P* < 0.001; *CP + HP distant*, *t* = −10.064, df = 99, *P* < 0.001; Fig. 2). On the other hand, treatments with *HP* from congeners lead to lower probabilities of pollen germination (<45 %), being worse than *CP* alone (*HP close*, *t* = 9.954, df = 99, *P* < 0.001; *CP + HP close*, *t* = 13.199, df = 99, *P* < 0.001; Fig. 2). At the tip of the style, the only treatment that had a similar probability of pollen tubes as *CP* was the one containing the mix of *CP + HP distant* (*t* = −1.375, df = 99, *P* = 0.645), while all other treatments had lower probabilities (Fig. 2). This pattern remained when we looked at pollen tubes in the base of the style (*CP + HP distant*, *t* = −2.453, df = 99, *P* = 0.110; Fig. 2).

### Recipient *Fuchsia regia* (specialist-pollination)

For this species, it is worth noticing that *CP* grains had the lowest germination probability on the stigma of all treatments (27 %), being different from both treatments containing *HP* from congeners (*HP close*, *t* = −5.371, df = 64, *P* = 0.013 and *CP + HP close*, *t* = −15.625, df = 64, *P* < 0.001), and also from *CP + HP distant* (*t* = −14.837, df = 64, *P* < 0.001; Fig. 2). Once pollen tubes entered the style, all treatments had similar probabilities as *CP* alone (20 %), except *HP distant* (with only 2 %, *t* = 5.333, *P* < 0.001; Fig. 2). All treatments end up showing similar probabilities than *CP* (that itself had a small probability, only 7 %) of having pollen tubes in the end of the style (*HP close*, *t* = 0.543, df = 63, *P* = 0.982; *CP + HP close*, *t* = 2.526, df = 63, *P* = 0.098; *HP distant*, *t* = 1.895, df = 63, *P* = 0.331 and *CP + HP distant*, *t* = −0.891, df = 63, *P* = 0.899; Fig. 2).

### Recipient *S. wettsteinii* (generalist-pollination)

All treatments had high probabilities of pollen germination on the stigma (75–99 %; Fig 2), but only *CP + HP distant* was high as *CP* (*t* = −0.738, df = 132, *P* = 0.947). Within the tip of the style, most treatments had lower probabilities than *CP*, except *CP + HP distant* that had a greater probability (*t* = −5.822, df = 132, *P* ≤ 0.001; Fig. 2). Once pollen tubes reached the base of the style, the treatment containing only *HP distant* continued to show a lower probability relative to *CP* (*t* = 6.396, df = 132, *P* < 0.001) and both treatments containing *HP close* had similar probabilities to *CP* (*HP close*, *t* = 0.879, df = 132, *P* = 0.904; *CP + HP close*, *t* = 0.022, df = 132, *P* = 1.0). At this point, *CP + HP distant* continued to perform better than *CP* alone (*t* = −5.252, df = 132, *P* < 0.001), with ca. 23 % greater chance of pollen tubes reaching the base of the style (Fig. 2).

## Discussion

Our results reinforce the idea that when species from the same genus are interacting via pollination, *HP* has a stronger negative impact on recipient (Ashman and Arceo-Gómez 2013; Arceo-Gómez and Ashman 2016). However, when the interaction is between distantly related species, recipients can tolerate or even benefit at the pollen tube stage from sharing pollinators with heterospecifics. As phylogenetic identity of *HP* source affects responses, the contribution of pollination systems was conditioned to that. Even though, our results indicate that both generalist- and specialist-pollinated species can tolerate *HP* in some level. The magnitude of *HP* response depends on the interactive effects between donor and recipient (Arceo-Gómez *et al.* 2019) and here we provide evidence that species phylogenetic relatedness is one of the factors involved in the complex equation of plant–plant post-pollination interactions.

### The effects of *HP* considering phylogenetic distance between donor and recipient

Even though we cannot differentiate which pollen tubes are from *HP close* and which ones are *CP* in the same style, our experiments using only *HP close* reveal that there are chances of hybridization between congeners. The lack of strong post-pollination barriers for *HP close*, as the ones that we present here, does not necessarily mean that these pollen tubes will fertilize the ovules. However, if the ovules are usurped by *HP* tubes this can be extremely costly to recipient plants since these ovules will no longer be available for *CP* tubes (Levin *et al.* 1996; Burgess *et al.* 2008). *HP close* tubes reach the base of the style in all species, but performances were different (greater or lesser probability of pollen-tube growth) depending on the specific treatment. We can check these differences by comparing the *CP + HP close* treatment with the treatments *CP* alone and *HP close* alone within each portion of the style. These comparisons allowed us to interpret which interactions modulated the final costs in each recipient species. In *S. wettsteinii*, the interaction between *CP* and *HP close* is competitive in stigmas, relaxing along the style since pollen tubes had similar probabilities of reaching style base in the three treatments. This possibly indicates a lack of incompatibility between these two species (*S. wettsteinii* and *S. glaziovii*), which might be a by-product of the recent speciation process of the genus (Chauveau *et al.* 2011). The competition is also strong during pollen germination in *F*. *campos-portoi*, but in this species it continues intense until the style base where both *CP + HP close* and *HP close* alone performed worse than *CP* alone. In *F. regia* stigmas, there is no apparent competition between *CP* and *HP close* germination; however, most pollen tubes of *CP + HP close* treatment are blocked prior to the first portion of style. Hence, their probabilities of success decrease from 64 % in stigmas to less than 1 % in style base, indicating that style strongly sieves pollen tubes. Considering how *Fuchsia* species impact each other reproduction, it is possible to see that when pollen tubes get in the style base, *F. regia* pollen plainly decreases *F. campos-portoi* post-pollination success. The reverse cross (*F. campos-portoi* donating pollen to *F. regia*) is more complex to interpret since all treatments (including the ones with *HP distant*) had slightly the same way, but based on the already mentioned substantial decrease of *CP + HP close* performance since arriving in stigma till style base, it also seems to be negative. Hence, it is likely that these responses are driven by an active mechanism (de Nettancourt 1977) rather than by incongruity since *CP* tubes are also being blocked. Nevertheless, further studies should consider applying genetic markers to check the strength of *HP close* in siring seeds in recipients, because some species might show a conspecific advantage over heterospecific in fertilizing ovules (Arnold 1997; Campbell *et al*. 2008) which then changes the signal of the interaction between closely related species to positive.

Heterospecific pollen distantly related (*HP distant*) germinated in all species when applied alone in stigmas, against general expectations (Martin 1970; Moreira-Hernández and Muchhala 2019). In fact, *HP distant* treatment had high probabilities of germination (especially in *F. campos-portoi*), which may suggest that in these species the stigmas themselves do not function to select pollen. This role seems to be played by the tip of the style where most of these pollen tubes are arrested, probably due to the lack of recognition resulted from the genetic distance (Hogenboom 1975). As they rarely got into the style (<4 %), this means that we observed mainly *CP* tubes in the final portion of styles in the treatment that combined *CP + HP distant*. Hence, *HP* can germinate even in phylogenetically distant species and have neutral (*Fuchsia* species) or even positive (*S. wettsteinii*) effects on recipient post-pollination success (pollen tubes in style end). A similar result was reported for *Cakile edentula* that when received *HP* of *Bidens pilosa* also increased *CP* tube growth, which was suggested to be stimulated by the release of biochemical compounds and result in a herd effect (Suárez-Mariño *et al.* 2019). In the context of pollination, the herd effect can be interpreted as the greater *CP* growth when in the presence of foreign pollen (*HP* in our case); however, this is an idea that has yet to be formally tested (Ashman *et al.* 2020). In this sense, when greater *CP* tubes number reach style base they are the result of recipient interaction with heterospecifics early in the stigma.

One important factor that might influence the extension of *HP* effect on distantly related species is how well pollen grains can adhere to recipients’ stigmatic surface. In the case of *S. wettsteinii* as recipient, only a small percentage of *O. confertissima* pollen adhered to its stigmas suggesting a weak attachment between the two species, which can be essential to avoid stigma blocking and its detrimental effects. Plus, the positive effect from their interaction could be due to their long history of coexistence. For instance, it has been shown that the opposite (new interactions between distantly related species) can lead to negative effects on recipient, like the ones provoked by only a few *Zea mays* pollen grains on *Mimulus guttatus* female fitness (Arceo-Gómez *et al.* 2018). Nevertheless, a previous study showed that other *Sisyrinchium* species (*S. campestre*) was not affected by a distantly related pollen donor (*Euphorbia esula*) invasive to the community (Montgomery 2009). These results taking together could indicate that species that encounter *HP* often in the evolutionary time (i.e. unrestrictive generalist-pollinated flowers) may have evolved mechanisms for tolerating it regardless of which species they are interacting with. However, whether the response is due to floral exposure to *HP* and detached from the history of coexistence with donors still needs to be formally tested.

### The effects of *HP* considering recipient’s pollination system

The role of pollination systems (i.e. specialized or generalized) determining the degree of plant response to *HP* remains an area in need of more study. Indeed, in here, recipients responded differently which might be associated with their historical exposure to *HP* but there was no consistent pattern within each category. *Sisyrinchium wettsteinii*, that has unrestrictive flowers visited by various groups of insects characterizing a generalist-pollination system (functional pollination *sensu*Ollerton *et al.* 2007), not only tolerate, as our hypothesis predicted, but can also respond positively to *HP*. On the other hand, both *Fuchsia* species, that show restrictive flowers pollinated by hummingbirds, being more specialized in the spectrum of plant–pollinator interactions (functional pollination *sensu*Ollerton *et al.* 2007), can also tolerate *HP* depending on the phylogenetic relatedness of donor. Hence, as our experiments were designed to assess the role of phylogenetic distance of *HP* relative to recipient, they might have hidden the real contribution of pollination systems.

### Additional traits potentially influencing recipient–donor interactions

Several floral traits besides phylogenetic distance are hypothesized to influence recipient–donor interactions affecting recipients’ responses. In the case of stigmatic surface, large stigmas capture more *HP* in nature (Montgomery and Rathcke 2012) but since there is still enough space for *CP* adherence, *HP* post-pollination effects seem to be minimum. Hence, we might expect that small stigmas are more negatively impacted by *HP* than larger ones. Interestingly, by our experiments it is possible to see the opposite when *HP distant* was in the arena. *Sisyrinchium wettsteinii* is the species with smallest stigmatic area in our set of recipients and was the only one that had its post-pollination success improved by the presence of *HP distant*. On the other hand, *HP close* seems to always have a negative effect regardless of recipient stigmatic area.

Pollen features like its size and apertures are also potential influencers of plant responses to *HP* (Ashman and Arceo-Gómez 2013). Our experiments indirectly suggest that other pollen feature that might affect the interaction as well is its water content. This was noticed due to *F. regia* low probabilities of pollen germination in *CP* treatment, which could be due to the application of a non-intentional amount of non-viable pollen. *Fuchsia* pollen grains were described as partially hydrated (high water content; Franchi *et al.* 2002) which means that they are fast germinators and, hence, strong competitors (Nepi *et al.* 2001). However, this condition makes them highly vulnerable to water loss, decreasing their viability rapidly after removed from anthers (Franchi *et al.* 2002), which possibly occurred in this specific treatment. Even happening in the treatment on which donor and recipient are the same species, this can be extended to heterospecific interactions. That is, we can hypothesize that partially hydrated pollen may not impair recipient post-pollination success as much as dry pollen due to its faster viability loss.

The distance between recipients’ stigmas and donors’ anthers may also influence *HP* effect in recipient since it represents interactors’ pollen flow. For instance, the different size of flowers of the two *Fuchsia* species may indicate that pollen flow between them is asymmetrical. *Fuchsia campos-portoi*, that has a smaller stigma height, has more chances of receiving *HP* from *F. regia* than the other way around. The former has some small chances of picking up *B. gounelleana* pollen from pollinator’s body while for the latter this is very unlikely to occur. Therefore, the long styles of *F*. *regia* are more effective as an avoidance mechanism to secure that few *HP close* pollen will reach stigmas. In the set of interactions with *S. wettsteinii* as recipient, the stigma–anther distance between this species and pollen donors is negligible, regardless of phylogenetic distance. Such absence of a mechanical barrier is likely because generalist-pollinated flowers usually do not show a mechanical fit with pollen vectors resulting in a pollen placed diffusely in pollinator’s bodies (Minnaar *et al.* 2019). The lack of specificity in pollen deposition and picking up could denote that conspecific pollination assurance is more relevant than *HP* costs, or basically that *HP* is not costly and can be even advantageous to recipient plants with generalist-pollinated systems, as we demonstrated in this case.

### Concluding remarks

Pollen performance was worst when *HP close* was applied to the stigmas. This reinforces that interactions via *HP* between congeners leads to more detrimental effects to recipients than when they get pollen from distantly related species (Arceo-Gómez and Ashman 2016). Nevertheless, our fine-scale study was able to demonstrate that plants can possibly also have positive reinforcements from receiving pollen of distantly related species. The evidence of tolerance and even benefits of *HP* in our experiments could be related to the community context that species are inserted. Tropical mountaintop communities, as the one here studied, are highly vulnerable to climatic variations, which makes the pollination environment very unpredictable (Freitas and Sazima 2006). For this plant community, it has been shown that species that flower nearby heterospecifics get more *CP* and grow more pollen tubes suggesting that the joint attraction of pollinators is advantageous under low pollinator availability circumstances (Bergamo *et al.* 2020a), but the role of *HP* receipt in such outcomes was not considered. Therefore, we hypothesize that the positive effect found when *S. wettsteinii* received *HP* distantly related in our study could be a reflex of the pollinator scarcity context, especially because this interspecific facilitation mentioned before was a trend among the generalist-pollinated species of this community (Bergamo *et al.* 2020a, b). Our results together with what is known from the studied community give new insights on how plant–plant post-pollination interactions may influence community assembly for further studies to explore.

## Data

All data and code are available at https://doi.org/10.6084/m9.figshare.c.4950777.v1
